# Supplementary material for: Competing charge-density wave instabilities in the kagome metal ScV6Sn6
Source: Nat Commun. 2023 Nov 23;14:7671. doi: 10.1038/s41467-023-43454-1 (PMC10667248; doi:10.1038/s41467-023-43454-1)
Supplement: Supplementary file 1 — SUPPLEMENTAL MATERIAL [file 41467_2023_43454_MOESM1_ESM.pdf]

Supplemental Information: Competing charge-density wave instabilities in the kagome metal ScV_6Sn_6

Saizheng Cao,[1] Chenchao Xu,[2] Hiroshi Fukui,[3] Taishun Manjo,[3]
Ying Dong,[4] Ming Shi,[5,1] Yang Liu,[1] Chao Cao,[1,*] and Yu Song[1,†]

[1]Center for Correlated Matter and School of Physics, Zhejiang University, Hangzhou 310058, China
[2]School of Physics, Hangzhou Normal University, Hangzhou 310036, China
[3]Japan Synchrotron Radiation Research Institute, SPring-8, 1-1-1 Kouto, Sayo, Hyogo 679-5198, Japan
[4]Research Center for Quantum Sensing, Zhejiang Lab, Hangzhou, 310000, People's Republic of China
[5]Photon Science Division, Paul Scherrer Institut, CH-5232 Villigen PSI, Switzerland

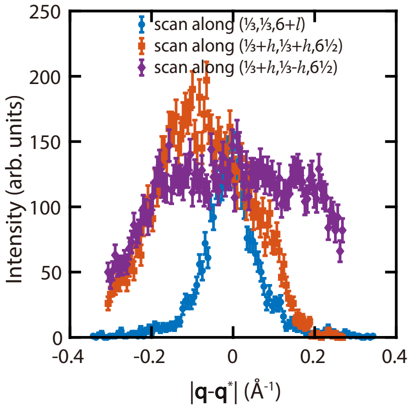

Supplementary Figure 1: Comparison of peak widths along three high-symmetry directions measured at 108 K. The blue, orange and purple symbols represent scans along $(\frac{1}{3}, \frac{1}{3}, 6+l)$, $(\frac{1}{3}+h, \frac{1}{3}+h, 6\frac{1}{2})$, and $(\frac{1}{3}+h, \frac{1}{3}-h, 6\frac{1}{2})$, respectively. The scans are plotted with the x-axis being $|\mathbf{q}-\mathbf{q}^*|$, in units of inverse angstroms. The error bars represent statistical errors of 1 s.d.

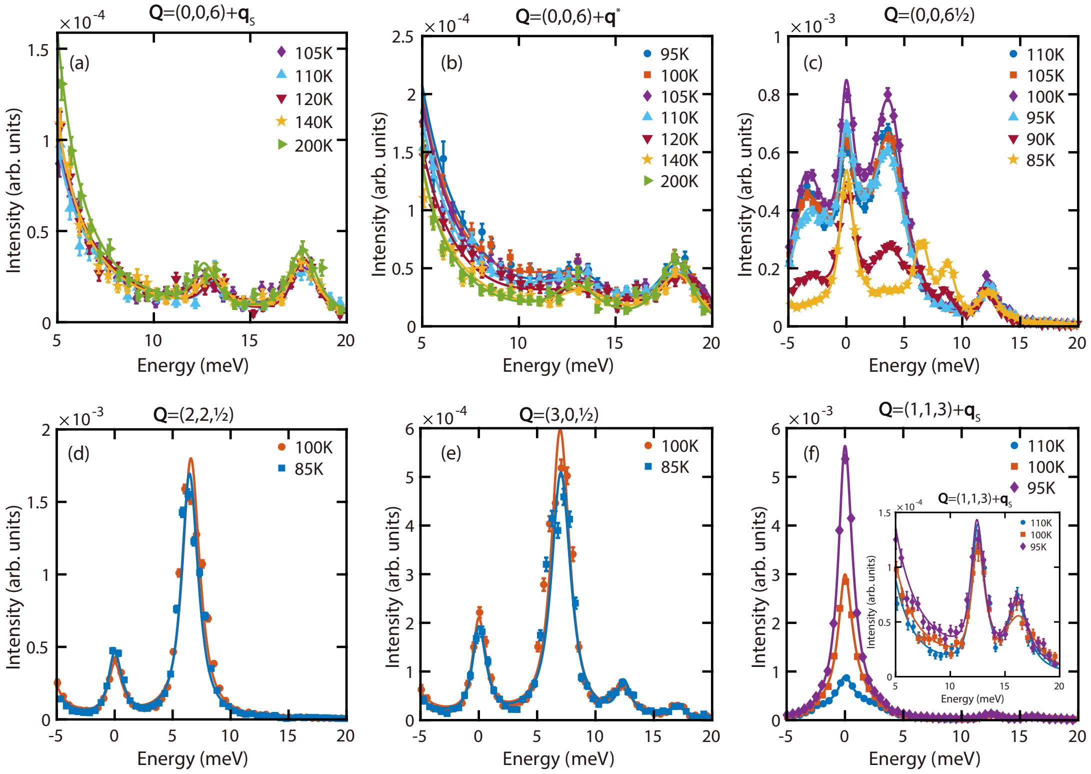

Supplementary Figure 2: Phonon spectra at various temperatures for wavevectors (a) $(0,0,6)+\mathbf{q}_\mathrm{s}$, (b) $(0,0,6)+\mathbf{q}^*$, (c) $(0,0,6\frac{1}{2})$, (d) $(2,2,\frac{1}{2})$, (e) $(3,0,\frac{1}{2})$, and (f) $(1,1,3)+\mathbf{q}_\mathrm{s}$. The solid lines are fits to Eq. 1, with n being the minimum number of phonon modes that can capture the measured data. We used either Eq. 2 or Eq. 3 for $S_i(E)$, depending on whether adding damping improves the fitting. The error bars represent statistical errors of 1 s.d.

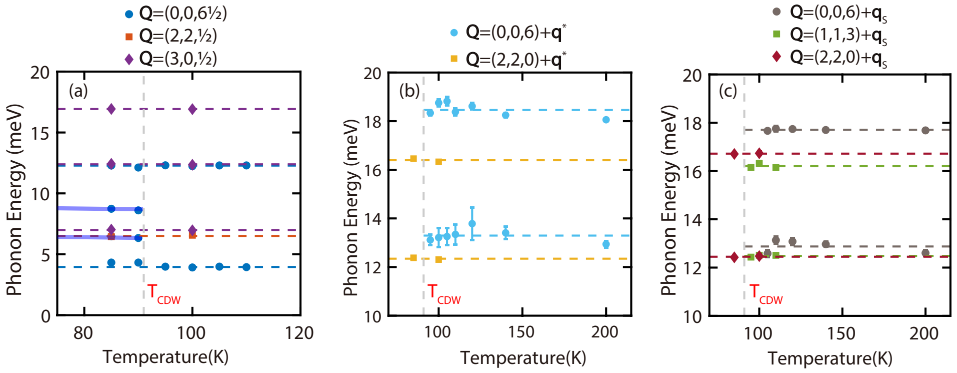

Supplementary Figure 3: Values of E_0 for (a) $\mathbf{q}=(0,0,\frac{1}{2})$, (b) $\mathbf{q}^*$, and (c) $\mathbf{q}_\mathrm{s}$, measured at various temperatures and in different Brillouin zones. The horizontal lines represent the mean values of E_0 at different temperatures. The thick lines represent phonon modes that appear below T_CDW. The error bars are least-square fit errors of 1 s.d.

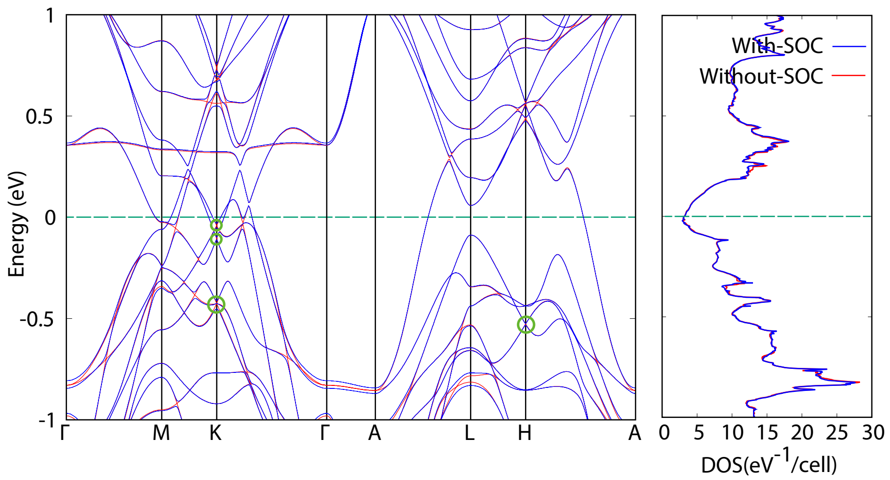

Supplementary Figure 4: Comparison of the calculated electronic band structures (left panel) and densities of states (right panel) of ScV_6Sn_6, with (blue) and without (red) spin-orbit coupling (SOC). Due to SOC, the massless Dirac cones at K and H become massive, see inside the green circles.

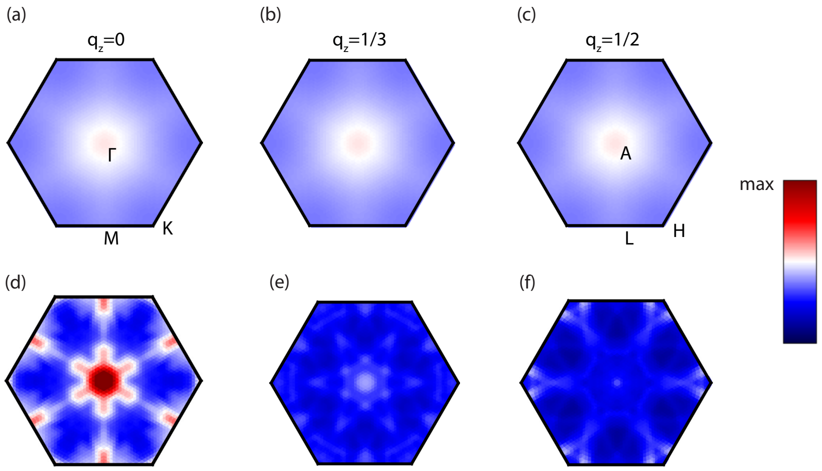

Supplementary Figure 5: The real part of the bare susceptibility (with SOC) in the static limit $\chi_0'(0, \mathbf{q})$, for (a) $q_z = 0$, (b) $q_z = \frac{1}{3}$, and (c) $q_z = \frac{1}{2}$ planes. The nesting function $J(\mathbf{q})$ (with SOC) for (d) $q_z = 0$, (e) $q_z = \frac{1}{3}$, and (f) $q_z = \frac{1}{2}$ planes.

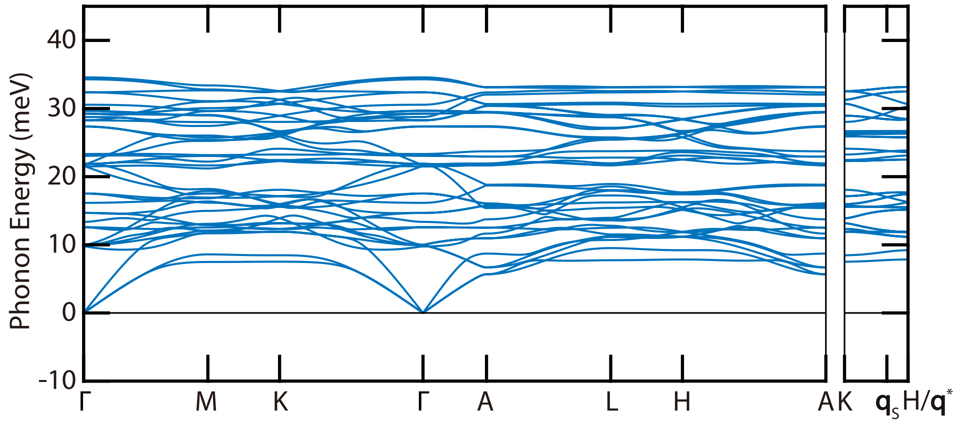

Supplementary Figure 6: The phonon spectrum of ScV_6Sn_6 calculated at a high electron temperature (∼0.1 Ry).

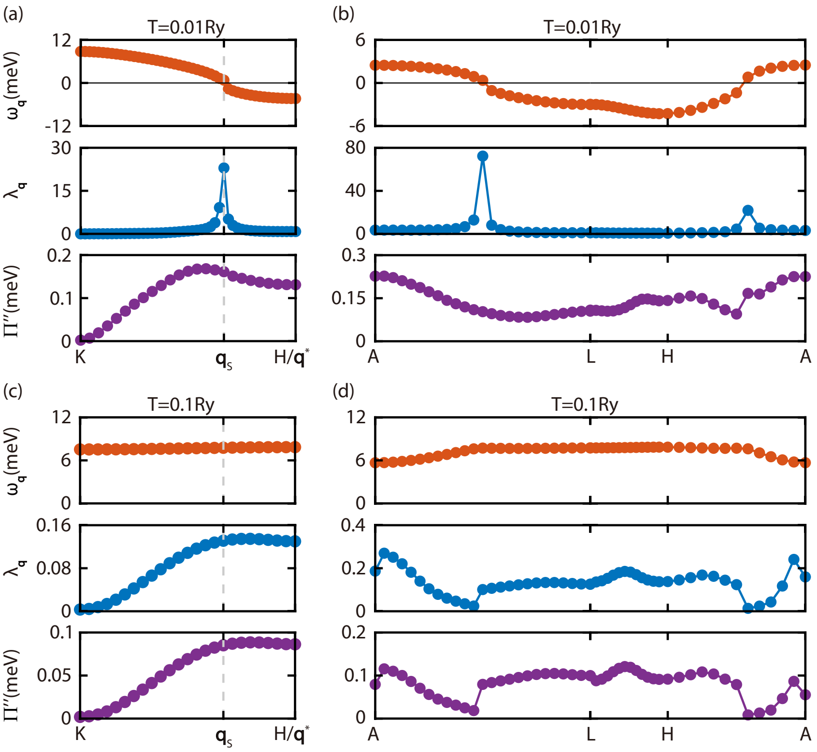

Supplementary Figure 7: Calculated phonon dispersion, EPC strength $\lambda_{\mathbf{q}\nu}$ and phonon self-energy $\Pi''_{\mathbf{q}\nu}$ at a low electron temperature ($\sim$0.01 Ry) along (a) $K-H$ and (b) $A-L-H-A$. Similar calculated results for a high electron temperature ($\sim$0.1 Ry) are shown in (c) and (d), respectively.

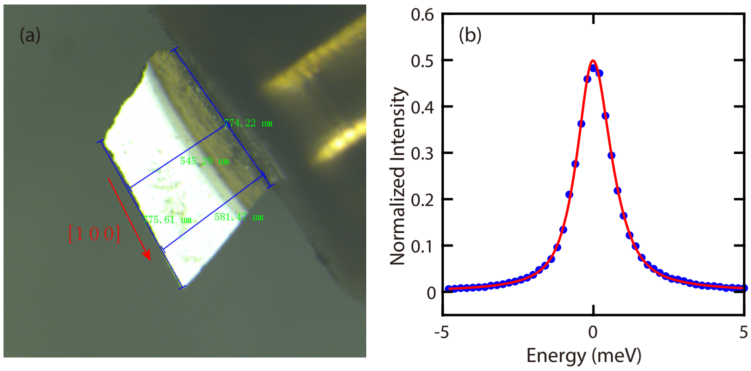

Supplementary Figure 8: (a) A photo of the ScV_6Sn_6 sample used in our inelastic X-ray scattering measurements. (b) The instrumental energy resolution $R(E)$ measured using a piece of PMMA, and is fit to a pseudo-Voigt function and normalized such that $\int_{-\infty}^{\infty} R(E)dE = 1$.

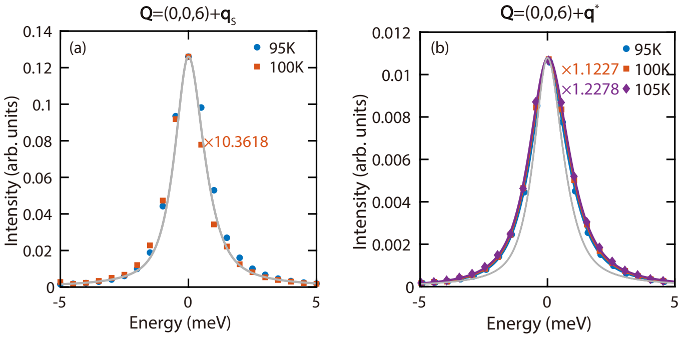

Supplementary Figure 9: Comparison of peaks centered around the elastic line at various temperatures for (a) $\mathbf{Q} = (0,0,6)+\mathbf{q}_s$ and (b) $(0,0,6) + \mathbf{q}^*$. The gray lines represent the energy resolution, and the solid lines with others colors in (b) are fits to Eq. 1. The data have been scaled so that their peak intensities match, allowing for a direct visual comparison of the peak widths. The error bars represent statistical errors of 1 s.d.

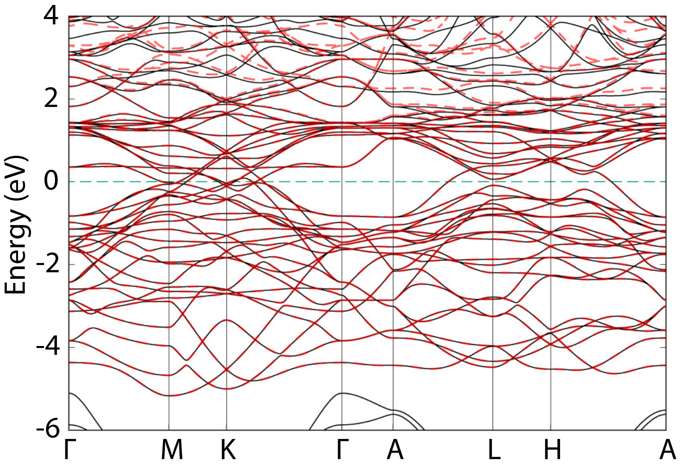

Supplementary Figure 10: The calculated electronic band structure from DFT without SOC (solid black lines), compared with the Wannier interpolation in `EPW` calculations (dashed red lines).

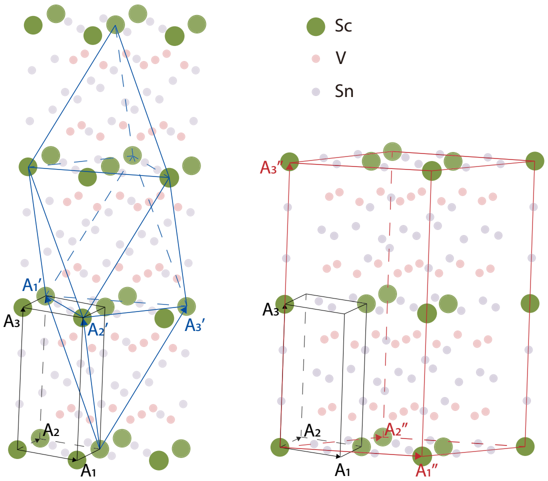

Supplementary Figure 11: Comparison between the $P6/mmm$ structure (lattice vectors $\mathbf{A}_1$, $\mathbf{A}_2$, and $\mathbf{A}_3$), with the rhombohedral primitive unit cell of $\mathbf{q}_s$-CDW (lattice vectors $\mathbf{A}'_1$, $\mathbf{A}'_2$, and $\mathbf{A}'_3$), and the hexagonal primitive unit cell of $\mathbf{q}^*$-CDW (lattice vectors $\mathbf{A}''_1$, $\mathbf{A}''_2$, and $\mathbf{A}''_3$).

Supplementary Note 1: Comparison of $\mathbf{q}^*$-CDW along orthogonal directions

To examine the peak profile of the short-range $\mathbf{q}^*$-CDW along different directions, scans at 108 K along $(\frac{1}{3}, \frac{1}{3}, 6+l)$, $(\frac{1}{3}+h, \frac{1}{3}+h, 6\frac{1}{2})$, and $(\frac{1}{3}+h, \frac{1}{3}-h, 6\frac{1}{2})$ are compared in Supplementary Fig. 1. Here, the measured momentum is $\mathbf{Q} = (0,0,6)+\mathbf{q}$, the scans are centered around $\mathbf{Q}^* = (0,0,6)+\mathbf{q}^*$, and the x-axis is the relative distance of $\mathbf{Q}$ away from $\mathbf{Q}^*$ ($|\mathbf{Q}-\mathbf{Q}^*| = |\mathbf{q}-\mathbf{q}^*|$), in units of inverse angstroms. As can be seen, $\mathbf{q}^*$-CDW is sharpest along l, and can be reasonably captured by a Lorentzian function. On the other hand, $\mathbf{q}^*$-CDW is more diffuse along $(h,h,0)$ and $(h,-h,0)$, and deviate significantly from the Lorentzian function. We note that while the $(h,h,0)$ scan appears to be off-center from $\mathbf{q}^*$, it likely results from the variation in structure factors of the associated soft phonons in different Brillouin zones. This is because $\mathbf{q}^*$ (H) is a high-symmetry point, which mandates the energies and damping rates of the phonon modes to be symmetric around it along $(h,h,0)$. However, there is no such requirement for the phonon structure factors.

Supplementary Note 2: Additional phonon measurements

As discussed in the Methods sections of the main text, the measured experimental phonon intensities are fit to the expression:

$$I(E) = b + cR(E-\delta E) + \sum_{i=1}^{n} \int_{-\infty}^{\infty} [S_i(E-\delta E-E')]R(E')dE', \tag{1}$$

and depending on whether a phonon is resolution-limited, $S_i(E)$ is:

$$S_i(E) = \frac{A_i}{1-\exp(-\frac{E}{k_\mathrm{B}T})} \frac{2}{\pi} \frac{\gamma_i E}{(E^2-E_{0i}^2)^2+(E\gamma_i)^2}, \tag{2}$$

or:

$$S_i(E) = \frac{A_i}{1-\exp(-\frac{E}{k_\mathrm{B}T})} \frac{\delta(E-E_{0i})-\delta(E+E_{0i})}{E}. \tag{3}$$

For $\mathbf{Q} = (0,0,6)+\mathbf{q}_\mathrm{s}$, data were measured in the range [-5,20] meV, with n determined to be 3 in Eq. 1 for energies up to 20 meV. In Fig. 3(a) of the main text, only data in the range [-5,5] meV with the lowest-lying soft mode are presented. Two additional phonon modes at higher energies are shown in Supplementary Fig. 2(a). The phonon mode around 13 meV is resolution-limited, and Eq. 3 is used in the fitting. The other two phonon modes are fit using Eq. 2. Measurements at $\mathbf{q}_\mathrm{s}$ are also carried out in the (220) and the (113) Brillouin zones, whereas soft phonons are hard to detect in the (220) Brillouin zone [Fig. 3(e) of the main text], they are detectable in the (113) Brillouin zone [Supplementary Fig. 2(f)]. Two phonon modes that hardly change in energy between 95 K and 110 K are also detected in the (113) Brillouin zone [inset in Supplementary Fig. 2(f)].

For $\mathbf{Q} = (0,0,6)+\mathbf{q}^*$, data in the range [-5,20] meV at various temperatures were fit to Eq. 1 with $n=3$ [Fig. 3(b) of the main text and Supplementary Fig. 2(b)]. The two phonon modes at higher energies (around 13 meV and 18 meV) are fit using Eq. 2.

For $\mathbf{Q} = (0,0,6)+(0,0,\frac{1}{2})$, the data above T_CDW can be fit to Eq. 1 using $n=2$ for energies up to 20 meV [Supplementary Fig. 2(c)]. Below T_CDW (85 K and 90 K), two additional phonon modes emerge, at energies around 6.4 meV and 8.7 meV. Interestingly, while the energies of these modes change little between 90 K and 85 K, their intensities increase significantly upon cooling. Similarly, the energy of the 4 meV mode hardly changes with temperature, but its intensity drops significantly upon cooling from 95 K and 85 K. These behaviors are consistent with a first-order $\mathbf{q}_\mathrm{s}$-CDW transition, with the volume fraction (reflected through changes in phonon intensities) changing with temperature, rather than the magnitude of the associated lattice distortion (reflected in the phonon energies). Additional measurements at $\mathbf{q} = (0,0,\frac{1}{2})$ were carried out in the (220) and (300) Brillouin zones [Supplementary Figs. 2(d) and (e)], revealing phonons modes that hardly changes across T_CDW (85 K and 100 K). These data were fit using Eq. 1 with $n=1$ and $n=3$, respectively. The 7 meV mode at $(3,0,\frac{1}{2})$ is not well-captured by a single DHO [Supplementary Fig. 2(e)], suggesting that there may be two phonon modes that are very close to each other around 7 meV.

Aside from the phonons that soften upon cooling [Figs. 3(a) and (b) in the main text and Supplementary Fig. 2(f)] and the two new modes that appear in the $\mathbf{q}_\mathrm{s}$ state [Supplementary Fig. 2(c)], all the other measured phonon modes

do not change significantly with temperature [Figs. 3(e) and (f) in the main text and Supplementary Fig. 2]. The fit values of E_0 for these temperature-independent phonon modes are summarized in Supplementary Figs. 3(a)-(c), at different $\mathbf{q}$-positions and in different Brillouin zones. The mean values of phonon energies at different temperatures [dashed lines in Supplementary Figs. 3(a)-(c)] are summarized and compared with DFPT calculations for $\mathbf{q}^*$ and $\mathbf{q} = (0, 0, \frac{1}{2})$ in Fig. 4(d) of the main text, and in Supplementary Fig. 3(d) for $\mathbf{q}_\mathrm{s}$. The standard deviations of phonon energies at different temperatures are shown as error bars.

Supplementary Note 3: Effects of spin-orbit coupling in first-principles calculations

The band structures and density of state (DOS) were calculated both without and with the spin-orbit coupling (SOC). As illustrated in the Supplementary Fig. 4(a), several Dirac points typical of the kagome lattice are identified at K (∼-0.1 eV and -0.04 eV) and H (∼-0.5 eV), marked with green circles. Once the SOC is considered, these Dirac points become gapped. The corresponding DOS is presented in Supplementary Fig. 4(b), revealing minor differences between calculations without and with the SOC, which indicate the SOC has a small effect on the electronic structure of ScV_6Sn_6.

In Supplementary Figs. 5(a)-(c), the real part of the bare electronic susceptibility in the static limit ($\chi_0'(0, \mathbf{q})$) are shown for $q_z = 0$, $q_z = \frac{1}{3}$ and $q_z = \frac{1}{2}$ planes, with SOC included in the calculations. $\chi_0'(0, \mathbf{q})$ is essentially featureless for all three q_z planes, and similar results were found in calculations that did not consider the SOC [1].

The nesting function $J(\mathbf{q})$ (with SOC) in the $q_z = 0$, $q_z = \frac{1}{3}$ and $q_z = \frac{1}{2}$ planes are illustrated in Supplementary Figs. 5(d)-(f). As can be seen, the nesting function with SOC exhibits almost the same features as the nesting function without SOC (shown in Fig. 4(c) of the main text). This confirms that SOC has a negligible effect on the bare electronic susceptibility, consistent with SOC having a minor effect on the electronic band structure and the DOS of ScV_6Sn_6 [Supplementary Fig. 4].

Supplementary Note 4: Comparison of phonon calculations at different electronic temperatures

In this section we compare the phonon dispersion $\omega_{\mathbf{q}\nu}$, electron-phonon coupling (EPC) $\lambda_{\mathbf{q}\nu}$ and phonon self-energy $\Pi''_{\mathbf{q}\nu}$ at low (∼0.01 Ry) and high (∼0.1 Ry) electron temperatures. Different electron temperatures of the phonon calculations were simulated by varying the Gaussian smearing in the DFT calculations.

For the low electron temperature (∼0.01 Ry), the phonon dispersion, $\lambda_{\mathbf{q}\nu}$ and $\Pi''_{\mathbf{q}\nu}$ for the phonon branch with lowest energy ($\nu = 1$) are shown in Supplementary Figs. 7(a) and (b), respectively along paths $K - H$ and $A - L - H - A$. Although peaks are observed along $K - H$ (at $\mathbf{q}_\mathrm{s}$), $A - L$ and $H - A$, a broad hump in $\Pi''_{\mathbf{q}\nu}$ is only observed along $K - H$ close to $\mathbf{q}_\mathrm{s}$. Since $\Pi''_{\mathbf{q}\nu}$ is the imaginary part of the phonon self-energy within the Migdal approximation, which is also directly associated with EPC, the absence of peak or hump features at positions where $\lambda_{\mathbf{q}\nu}$ peak along $A - L$ and $H - A$ suggest these peaks in $\lambda_{\mathbf{q}\nu}$ are likely artifacts of $\omega_{\mathbf{q}\nu}$ crossing zero. On the other hand, a broad hump in $\Pi''_{\mathbf{q}\nu}$ around $\mathbf{q}_\mathrm{s}$ evidences an enhanced wavevector-dependent EPC around $\mathbf{q}_\mathrm{s}$, which likely favors selection of the $\mathbf{q}_\mathrm{s}$-CDW as the ground state. This is consistent with the hump in $\Pi''_{\mathbf{q}\nu}$ being more prominent at the low electron temperature [Supplementary Figs. 7(a) and (c)].

For the high electron temperature (∼0.1 Ry), the calculated phonon spectrum is free of imaginary phonons, as shown in Supplementary Fig. 6. The phonon dispersion, $\lambda_{\mathbf{q}\nu}$ and $\Pi''_{\mathbf{q}\nu}$ for the phonon branch with lowest energy ($\nu = 1$) are shown in Supplementary Figs. 7(c) and (d), respectively along paths $K - H$ and $A - L - H - A$. A broad hump centered around $\mathbf{q}_\mathrm{s}$ is observed in $\lambda_{\mathbf{q}\nu}$ along $K - H$, this further corroborates the presence of an enhanced wavevector-dependent EPC around $\mathbf{q}_\mathrm{s}$. On the other hand, minima in $\lambda_{\mathbf{q}\nu}$ are observed along $A - L$ and $H - A$, at positions where $\lambda_{\mathbf{q}\nu}$ peak at the low electron temperature.

In summary, our calculations in Supplementary Fig. 7 reveal (1) a $\Pi''_{\mathbf{q}\nu}$ hump around $\mathbf{q}_\mathrm{s}$ that becomes enhanced at the low electron temperature, and (2) a $\lambda_{\mathbf{q}\nu}$ broad peak around $\mathbf{q}_\mathrm{s}$ at the high electron temperature. Both behaviors indicate an enhanced $\mathbf{q}$-dependent EPC around $\mathbf{q}_\mathrm{s}$. On the other hand, the absence of these features where $\lambda_{\mathbf{q}\nu}$ peaks along $A - L$ and $H - A$ at the low electron temperature, suggest these peaks in $\lambda_{\mathbf{q}\nu}$ are likely artifacts due to $\omega_{\mathbf{q}\nu}$ crossing zero.

---

* Electronic address: ccao@zju.edu.cn

† Electronic address: yusong_phys@zju.edu.cn

[1] Tan, H. & Yan, B. Abundant lattice instability in kagome metal scv_6sn_6. *Phys. Rev. Lett.* **130**, 266402 (2023).
